# Lecanemab in the treatment of early Alzheimer's disease: An observational study based on 7.0T ultra‐high field MRI

**DOI:** 10.1002/alz70856_100048

**Published:** 2025-12-25

**Authors:** Haokun Liu, Weiwei Zhang, Keying Fang, Bin Jiao, Lu Shen

**Affiliations:** ^1^ Xiangya Hospital, Central South University, Changsha, Hunan, China

## Abstract

**Background:**

Previous research has demonstrated the effectiveness of lecanemab in treating early Alzheimer's disease (AD). However, its validation in the Hunan region of China remains limited. Traditional evaluation methods lack precision, particularly in brain imaging technologies. To address this gap, this study utilizes 7.0‐tesla ultra‐high‐field magnetic resonance imaging (MRI) and its sensitive susceptibility‐weighted imaging (SWI) sequence, which effectively detects microbleeds. Additionally, diffusion tensor imaging (DTI) analysis, in combination with the along the perivascular spaces (ALPS) index, is employed to evaluate glymphatic function. This study aims to assess the efficacy and safety of lecanemab in AD treatment, focusing on its impact on brain microstructure, glymphatic function, and amyloid‐β (Aβ) clearance.

**Method:**

Beginning on June 28, 2024, 27 patients diagnosed with AD were enrolled in the study and commenced treatment with lecanemab. Comprehensive baseline data collection, clinical screening, and cognitive assessments were completed. Each patient underwent SWI and DTI imaging using a 7.0T Siemens Terra MRI scanner equipped with a 32‐channel head coil and parallel transmission (pTX) system, both pre‐ and post‐treatment.

**Result:**

Of the 27 participants (mean age 61.67±7.30 years; 9 male), two patients (7.4%; 1 male) experienced flu‐like symptoms following their initial lecanemab injection. No new cortical or subcortical microhemorrhages were detected via 7.0T MRI SWI sequences during treatment. Additionally, there were no instances of ARIA‐E or ARIA‐H among participants. Cognitive function, evaluated through the Mini‐Mental State Examination (MMSE) and Montreal Cognitive Assessment (MoCA), showed no significant decline during the observation period (*p* >0.05). Among the 27 patients, 9 (mean age 63.56±8.90 years; 4 male) completed observation for their seventh medication dose. Paired t‐tests revealed a statistically significant increase in left DTI‐ALPS index values post‐treatment (t = ‐2.781, *p* =  0.024), suggesting improvements in glymphatic function.

**Conclusion:**

1. Lecanemab treatment in AD patients from Hunan, China, is generally safe, though its long‐term efficacy requires further investigation. 2. The use of 7.0T MRI, particularly its SWI sequence, enhances monitoring of microbleeds and improves safety surveillance. Furthermore, DTI‐ALPS, which reflects the glymphatic function, presents as a potential imaging marker for assessing treatment efficacy.

